# Enhancing immune regulation *in vitro*: the synergistic impact of 3′-sialyllactose and osteopontin in a nutrient blend following influenza virus infection

**DOI:** 10.3389/fimmu.2024.1271926

**Published:** 2024-02-15

**Authors:** Zhengtao Guo, Qinggang Xie, Qiqi Ren, Yang Liu, Kaifeng Li, Bailiang Li, Jufang Li

**Affiliations:** ^1^School of Food, Northeast Agricultural University, Harbin, Heilongjiang, China; ^2^Key Laboratory of Dairy Science, College of Food Science, Northeast Agricultural University, Harbin, Heilongjiang, China; ^3^Feihe Reseach Institute, Heilongjiang Feihe Dairy Co., Beijing, China

**Keywords:** 3′-sialyllactose, osteopontin, influenza virus, immune cytokines, human milk oligosaccharide

## Abstract

Natural components of breast milk, human milk oligosaccharides (HMOs) and osteopontin (OPN) have been shown to have a variety of functional activities and are widely used in infant formulas. However, the preventive and therapeutic effects of both on influenza viruses are not known. In this study, antiviral assays using a human laryngeal carcinoma cell line (HEP-2) showed that 3′-sialyllactose (3′-SL) and OPN had the best antiviral ability with IC_50_ values of 33.46 μM and 1.65 μM, respectively. 3′-SL (10 μM) and OPN (4 μM) were used in combination to achieve 75% inhibition. Further studies found that the combination of 200 μg/mL of 3′-SL with 500 μg/mL of OPN exerted the best antiviral ability. The reason for this was related to reduced levels of the cytokines TNF-α, IL-6, and iNOS in relation to mRNA expression. Plaque assay and TCID_50_ assay found the same results and verified synergistic effects. Our research indicates that a combination of 3′-SL and OPN can effectively reduce inflammatory storms and exhibit anti-influenza virus effects through synergistic action.

## 1 Introduction

Influenza is a respiratory disease that spreads easily and causes symptoms such as a runny nose, sore throat, fever, and in severe cases, pneumonia and complications in other organs (1). Scientific data reveals that influenza viruses are responsible for hundreds of millions of infections worldwide annually, with infants and children particularly vulnerable due to their weakened immune system. In 2018, there were nearly 109.5 million reported cases of viral infections among children under the age of 5 and approximately 34,800 deaths attributed to influenza or its complications globally (2). These figures demonstrate the significant threat that influenza viruses pose to human health, particularly in young children. While antiviral drugs currently play a crucial role in treating influenza. However, ethical concerns and strict regulations surrounding clinical trials in infants and children have restricted access to appropriate antiviral medications for this demographic (3, 4), Moreover, the high mutagenicity of influenza viruses has led to reduced efficacy of vaccines, further limiting treatment options (5). Indeed, there is an urgent need for new approaches to improve infants and childrens resistance to viruses.

Breast milk is widely recognized as the optimal food for infants, offering adequate nutrition as well as numerous active ingredients that promote anti-inflammatory (6), anti-infective (7), and immune development in early infancy (8). Human milk oligosaccharides (HMOs), the third most abundant component in human milk, confer important benefits for early colonization of the infants gut microbiota, intestinal barrier function, and immune modulation (9). HMOs have been shown to have unique antiviral properties and some studies have demonstrated their potential in reducing the risk of infection from a wide range of viruses. Clinical evidence supports the idea that HMOs have the ability to withstand the harsh conditions in the stomach and anterior small intestine. This enables them to reach the distal small intestine where they can stabilize G10P rotaviruses that infect cells (10). In a controlled dietary model of rotavirus-induced diarrhea in piglets, the experimental group received milk powder with HMOs showed a significant reduction in the duration of diarrhea compared to the control group (11). The 2-fucosyllactose (2-FL) mimics the human norovirus receptor, the histo-blood group antigen (HBGA), and acts as a decoy to prevent norovirus binding to HBGA, ultimately reducing the symptoms of infection (12). Osteopontin (OPN) is a highly abundant multifunctional non-collagenous matrix phosphoprotein in breast milk (13, 14). Research has demonstrated that OPN exhibits antiviral properties, and that mice with impaired OPN gene expression showed reduced immunity to viral and bacterial infections (15). Furthermore, a recent study discovered that both 2-FL and OPN were highly effective in reversing DNCB-induced dermatitis in mouse models, with an even more pronounced restorative effect observed when the two compounds were used together (16). In summary, the active components found in breast milk, particularly HMOs and OPN, have shown remarkable antiviral properties and may potentially exhibit synergistic effects. These findings provide strong motivation for further research into their effectiveness against influenza viruses and for studying the unique impact they can have when used together.

We utilized 2′-fucosyllactose (2′-FL), 3′-sialyllactose (3′-SL), 6′-sialyllactose (6′-SL), lacto-N-tetraose (LNT), lacto-N-neotetraose (LNnT), and OPN to intervene in an *in vitro* experiment of human laryngeal cancer cells infected with H1N1 influenza virus. Our aim was to investigate the potential therapeutic effects of these compounds on infected cells. The most effective HMO was then selected for combination with OPN to observe their synergistic effects and explore possible mechanisms of influence. Our objective is to propose a new personalized approach for infants and children with influenza virus, distinct from conventional drug treatment.

## 2 Materials and methods

### 2.1 Cells and strains

The HEP-2 human laryngeal carcinoma cell line was obtained from the American Type Culture Collection (ATCC) and cultured in modified DMEM medium (GibcoTM Life Technologies Inc., Grand Island, USA), supplemented with 10% fetal bovine serum (FBS, GibcoTM Life Technologies Inc., Grand Island, USA) and 1% dual antibodies (10,000 U/mL penicillin-10,000 g/mL streptomycin, Solarbio life sciences, Beijing, China). Influenza A (H1N1) virus (A/Zhejiang-Kecheng/SWL1219/2023) was provided by the Chinese Centre for Disease Control and Prevention and stored at -80°C. The virus was passaged twice through chicken embryos before the experiment to determine its potency for use, the first titer is 2^-6^, the second is 2^-7^, and the virus with the smaller titer is taken for follow up experiments. TNF-α, iNOS, and IL-6 ELISA kits were obtained from MULTISCIENCES (Shenzhen, China).

### 2.2 HMOs and OPN

This study utilized five different breast milk oligosaccharides (HMOs), with purities exceeding 95%, purchased from Royal DSM Group in the Netherlands. These HMOs included one fucose-based neutral oligosaccharide, 2′-fucosyllactose (2′-FL), two non-fucose-based neutral oligosaccharides, lacto-N-tetraose (LNT) and lacto-N-neotetraose (LNnT), and two acidic oligosaccharides, 3′-sialyllactose (3′-SL) and 6′-sialyllactose (6′-SL). Additionally, Lacprodan OPN-10 bone bridging protein with a purity level of over 95% was provided by Arla Foods Ingredients. The HMOs were dissolved in a 20 mM master batch using dimethyl sulfoxide (DMSO, Sigma, USA), while the OPN was dissolved in a 2 mg/mL master batch using sterile PBS for use. The OPN and HMOs used in the study were extracted and purified from bovine milk or synthesized using methods such as enzymatic reaction and microbial fermentation to obtain humanized osteopontin. Both production and use have been certified by the European Food Safety Authority (EFSA).

### 2.3 H1N1 virus TCID_50_ analysis

A frozen solution of the H1N1 influenza virus was diluted in a 10-fold gradient with a serum-free DMEM medium. The diluted virus solution (100 μL/well) was added to 96-well plates lined with monolayers of cells, with six replicate wells used for each concentration, and a control group of normal cells. The plates were incubated in a 5% CO_2_ incubator at 37°C for two hours, then the virus dilution solution was replaced with cell maintenance solution, and the plates were allowed to incubate for a further 48 hours, with the degree of cellular lesions and number of wells recorded by observing cell morphology. When cell controls were close to normal morphology, wells with ≥50% virus-infected cytopathic lesions were considered diseased wells. The amount of virus infection in half of the cell cultures (TCID_50,_ 50% tissue culture infective dose) was calculated using the Reed-Muench method.

### 2.4 Cytotoxicity analysis

HEP-2 cells were recovered and passaged 3-4 times until they grew well. The cells were then trypsin digested, diluted to a concentration of 1.5 x 10^5^ cells/mL with complete medium, mixed, and blown before being inoculated into 96-well plates at 100 μL/well. The plates were incubated at 37°C in a 5% CO2 incubator for 24 h. The substances being tested were serially diluted in pairs with culture medium, and each concentration was added to the 96-well plates at 100 μL/well, with three replicate wells for every concentration, and a blank control group was included. The cells were incubated at 37°C in a 5% CO_2_ incubator for 48 h. Afterward, cell morphology was observed and the supernatant was removed. CCK-8 was prepared as a 10% solution in PBS and 100 μL was added to each well. After one hour of action, the absorbance of the cells was measured using an enzyme marker at a wavelength of 540 nm.

### 2.5 Cytopathic inhibition assay

The culture medium was aspirated, and 100 TCID_50_ of virus solution (control and treatment groups) or culture medium (normal group) was added at 100 µL/well. After adsorbing in a 37°C, 5% CO_2_ incubator for 4 h, the influenza virus liquid was aspirated and 100 µL of compounds at appropriate doses were added to each well, with three replicate wells per concentration repeated three times. The cells were observed for 48 h to check for any morphological changes, and the cell supernatant was discarded. Next, CCK-8 was prepared as a 10% solution in PBS, and 100 μL was added to each well. After being given one hour to interact with the cells, enzymatic markers measured cell absorbance at a wavelength of 540 nm. Using the formula: Inhibition rate (%) = (Drug average A value - Virus control group average A value)/(Cell control average A value - Virus control group average A value) ×100%, calculate the degree of suppression of viral-induced cellular deterioration by the drug.

### 2.6 ELISA analysis

After a 4-hour adsorption period at 37°C in a 5% CO_2_ incubator, the influenza virus solution was aspirated, and 100 µL of compounds at varying doses were added to 3 replicate wells per concentration, which was repeated 3 times. Following a 48-hour incubation period, the supernatant was collected and analyzed using an ELISA kit to measure the levels of inflammatory factors, including TNF-α, iNOS, and IL-6.

### 2.7 RT-qPCR analysis

After adsorbing the influenza virus solution for 4 hours at 37°C in a 5% CO_2_ incubator, the solution was removed and replaced with 100 µL L of each compound at the appropriate dose across 3 wells per concentration, repeated 3 times. After 48 hours, the supernatant was removed, and cells were washed twice with pre-chilled PBS. Total RNA was extracted from the cells using Trizol according to the manufacturers instructions. Subsequently, mRNA expression levels of TNF-α, iNOS, and IL-6 inflammatory factors were measured by real-time fluorescence PCR after reverse transcription. GAPDH was used as an endogenous control, and the primers used in this study were: GADPH-Forward-(5-GACCCCTTCATTGACCTCAAC-3), GADPH-Reverse-(5-CATACCAGGAAATGAGCTTG-3), TNF-α-Forward-(5-CTGCTGCACTTTGGAGTGAT-3), TNF-α-Reverse-(5-AGATGATCTGACTGCCTGGG-3), IL-6-Forward-(5-AGCCACTCACCTCTTCAGAAC-3), IL-6-Reverse-(5-GCCTCTTTGCTGCTTTCACAC-3), iNOS-Forward-(5-CATCCTCTTTGCGACAGAGAC-3), iNOS-Reverse-(5-GCAGCTCAGCCTGTACTTATC-3). 2-ΔΔCTwas used to quantitatively analyze the mRNA of genes.

### 2.8 Inhibition of influenza H1N1 virus by the compositions

Plaque Assay: Inoculate HEP-2 cells into 6-well plates (1.5x10^5^/well) and incubate overnight at 37°C, 5% CO_2_. The culture medium was aspirated, and 100 TCID_50_ of virus solution (H1N1 and treatment groups) or culture medium (control group) was added at 1 mL/well. After adsorbing in a 37°C, 5% CO_2_ incubator for 4 h. Control group, 3′-SL group (200 μg/mL), OPN group (500 μg/mL), 3′-SL group (200 μg/mL), and 3′-SL+OPN group (200 μg/mL+500 μg/mL) were set up with the H1N1-infected group, adding 1mL of the compound to the corresponding wells. After that, each well was covered with a nutrient mixture containing 0.2% Bovine Serum Albumin (BSA, Beyotime, Shanghai, China), 0.6% Agar (Beyotime, Shanghai, China), and 0.3% DEAE (Beyotime, Beijing, China). The plates were then incubated at 37°C under 5% CO_2_ conditions for 2-3 days. Subsequently, the wells were fixed and stained using a 0.5% crystal violet solution with formalin, and the plaque was observed.

TCID_50_ Assay: A frozen solution of the H1N1 influenza virus was diluted in a 10-fold gradient with a serum-free DMEM medium. The diluted virus solution (100 μL/well) was added to 96-well plates lined with monolayers of cells, with six replicate wells used for each concentration, and a control group of normal cells. The plates were incubated in a 5% CO_2_ incubator at 37°C for two hours, then the virus dilution solution was replaced with cell maintenance solution, then 100 µL of each of the compounds at the appropriate dose was added. Incubate for 48 h to observe the lesions (CPE) after viral infection of the cells, and the TCID_50_ of the different drug concentration groups was determined by the Reed-Muench method.

### 2.9 Statistical analysis

The significance statistics were performed by One-way ANOVA analysis, Duncans multiple comparison test, and paired-samples T test of variance with SPSS 22 Version. A P value <0.05 was considered indicative of statistical significance. All experiments were repeated at least 3 times, and the data were expressed as mean ± standard deviation (Mean ± SD).

## 3 Result

### 3.1 Determination of TCID_50_ of H1N1 influenza virus

The influenza virus was passaged twice in SPF-grade chicken embryos, and the number of wells in which the H1N1 virus appeared cytopathic for each virus dilution at 42h was observed. The TCID_50_ value for the infection of cells by the H1N1 strain of influenza virus was calculated to be 10^-3.48^/0.1mL using the Reed-Muench method.

### 3.2 Toxic effects of HMOs and OPN on HEP-2 cells

**Figure 1:**
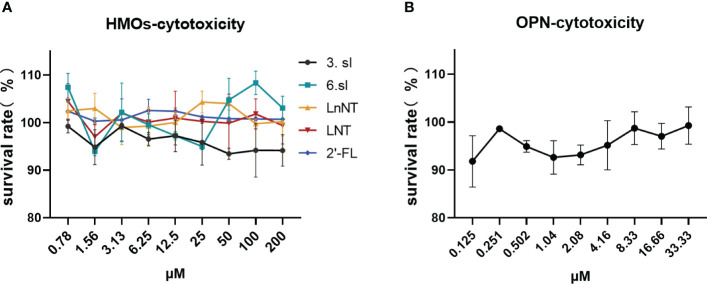
Toxic effects of HMOs and OPN on HEP-2 cells. **(A)** Toxic effects of five breast milk oligosaccharides on HEP-2 cells. **(B)** Toxic effects of bone bridge protein (OPN) on HEP-2 cells. Data are represented as mean ± SD (n= 3). Cell viability was calculated according to the formula: % cell viability = absorbance value of the administered group A/absorbance value of the cell control group A x 100%.

We first assessed the toxic effects of these six nutrients in HEP-2 cells before *in vitro* antiviral assays as a way to avoid using concentrations that are toxic to cells for antiviral assays. The six nutrients were found to have close to 100 percent cell survival whether administered at high or low concentrations (**Figures 1A, B**), suggesting that they have no significant effect on cell survival and are sufficiently safe.

### 3.3 Inhibitory effect of HMOs and OPN on HEP-2 cytopathy caused by H1N1 virus

To investigate whether these six nutrients could inhibit H1N1 influenza virus infection, antiviral assays were performed using HEP-2 cells and only OPN and 3′-SL were found to have a significant inhibitory effect on H1N1-infected cells., we calculated the IC_50_ values of six nutrients against the H1N1 virus using a non-linear fit (**Table 1**). The IC_50_ values for 3′-SL and OPN were 33.46 μM and 1.65 μM, respectively, while several others including fucosyl-neutral and non-fucosyl-neutral and other acidic oligosaccharides showed less than 50% inhibition at all concentrations, indicating that the anti-H1N1 influenza virus was not effective. Based on these results, we determined the excellent antiviral properties of OPN with 3′-SL.

**Table 1:** Results of nutrient inhibition of viral cytopathogenesis.

Nutrients	IC_50_
2′-FL	>200 μM
3′-SL	33.46 μM
6′-SL	>200 μM
LNT	>200 μM
LNNT	>200 μM
OPN	1.65 μM

### 3.4 Inhibition of viral HEP-2 cytopathic lesions by 3′-SL complex OPN

Preliminary antiviral experiments were conducted to assess the combined antiviral properties of 3′-SL+OPN. Based on the data, we observed inhibition rates of 40% and 70% for H1N1 at OPN concentrations of 1 μM and 4 μM, respectively, and inhibition rates of 45% and 60% at 3′-SL concentrations of 10 μM and 40 μM, respectively. A combination of OPN (4 μM) and 3′-SL (10 μM) demonstrated higher inhibition rates (75%) than the monomers at lower concentrations (**Table 2**), suggesting the need to investigate optimal concentration for inhibiting the pathogenic effect of 3′-SL in combination with OPN against H1N1 virus.

**Table 2:** Inhibition rate of viral cytopathogenic lesions by nutrients and their combinations.

OPN	concentration (μM)	1	4
	Inhibition rate (%)	40	70
3′-SL	concentration (μM)	10	40
	Inhibition rate (%)	45	60
OPN+3′-SL	concentration	4 (μM)+10 (μM)	
	Inhibition rate (%)	75.21 ± 6.15	

### 3.5 H1N1 influenza virus inhibition assay

**Figure 2:**
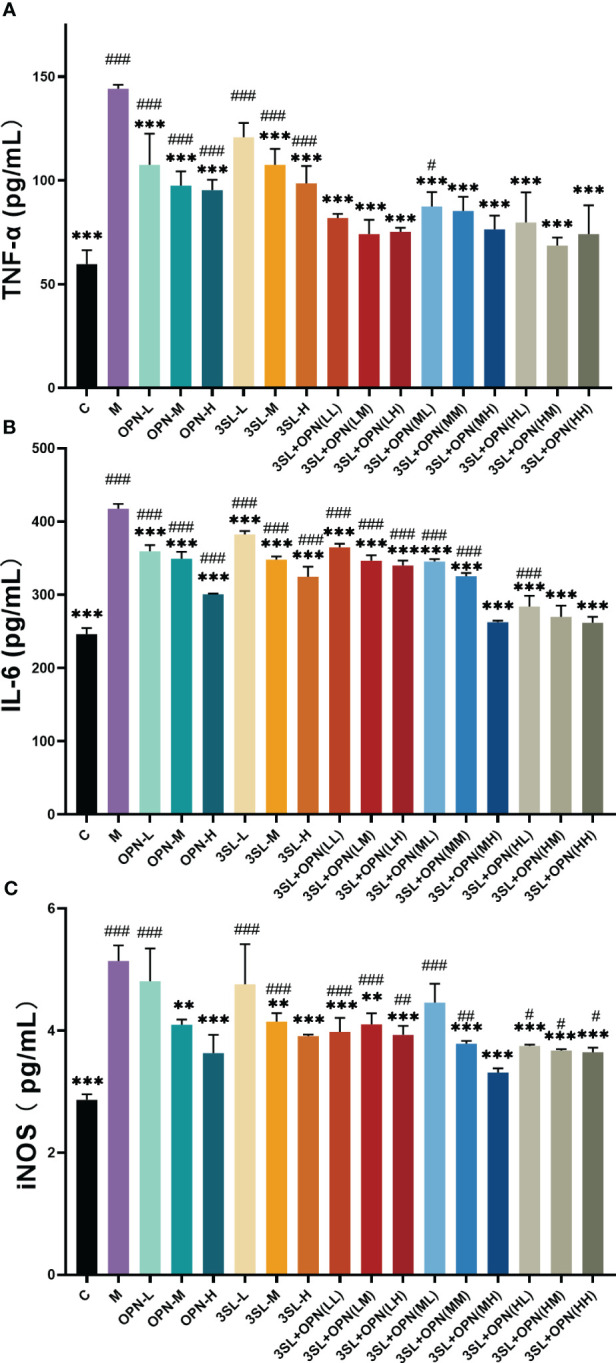
Effects of nutrient concentrations and combinations on suppression of inflammation following H1N1 infection. The study included the following groups: control group (C), a model group (M), and various groups treated with different doses of 3′-SL and OPN alone, including low dose (20 μg/mL, 3SL-L), medium dose (200 μg/mL, 3SL-M), and high dose (600 μg/mL, 3SL-H) of 3′-SL, and low dose (60 μg/mL, OPN-L), medium dose (200 μg/mL, OPN-M), and high dose (500 μg/mL, OPN-H) of OPN. 3′-SL+OPN group: (20 μg/mL + 60 μg/mL, LL), (20 μg/mL + 200 μg/mL, LM), (20 μg/mL + 500 μg/mL, LH), (200 μg/mL + 60 μg/mL, ML), (200 μg/mL + 200 μg/mL, MM), (200 μg/mL + 600 μg/mL, MH), (500 μg/mL + 60 μg/mL, HL), (500 μg/mL + 200 μg/mL, HM), and (500 μg/mL + 600 μg/mL, HH). **(A)** TNF-a, **(B) **IL-6, **(C)** iNOS. Data are represented as mean ± SD (n= 3). *P< 0.05; **P <0.01; ***P <0.001, compared with the Model group M. ^#^P <0.05; ^##^P< 0.01; ^###^P <0.001, compared with the control group C.

The impact of nutrients and their combinations on inflammatory factors was evaluated following infection of cells with the H1N1 virus, as illustrated in **Figures 2A–C**. The control group displayed levels of TNF-a, IL-6, and iNOS at 59.67 ± 6.67 pg/mL, 246.39 ± 8.24 pg/mL, and 2.87 ± 0.09 pg/mL, respectively. On the other hand, the model group showed a significant increase (p ≤ 0.001) in all three inflammatory factors after virus infection, with TNF-a, IL-6, and iNOS levels at 144.11 ± 1.92 pg/mL, 417.62 ± 6.46 pg/mL, and 5.14 ± 0.25 pg/mL, respectively. The results of this study suggest that the H1N1 virus upregulates pro-inflammatory factors, which can trigger an inflammatory storm and lead to tissue damage. After treatment with different doses of OPN and 3′-SL, a decrease in pro-inflammatory factors was observed. However, analysis of variance indicated that six different concentrations of the nutrients exhibited high sensitivity (p ≤ 0.001) in inhibiting IL-6 production compared to the model group. In contrast, for TNF-a, significant differences were only found at medium and high concentrations of 3′-SL in combination with three concentrations of OPN, while no significant differences were observed at low concentrations (p>0.05). As for iNOS production, significant differences were found at medium and high concentrations of both OPN and 3′-SL, but no significant differences were observed at low concentrations when compared to the model group (p>0.05). These findings indicate that OPN and 3′-SL have a potential therapeutic effect on inflammation induced by H1N1 influenza virus.

Combination of two nutrients, OPN and 3′-SL, demonstrates superior inhibition of inflammatory factors. Analysis of variance showed highly significant differences (p < 0.01) in IL-6 and TNF-a compared to the model group in all nine groups with different dose combinations of high, medium and low. Additionally, iNOS production was highly significantly different from the model group (p<0.001) in all combinations except for 3′-SL+OPN (ML). Based on these results, it can be concluded that the synergistic effect of combining the two nutrients is more potent in suppressing H1N1 virus-induced inflammation as compared to using either nutrient alone.

Further, a comparative analysis of the inhibitory effect of nine combinations on the cytopathogenic effect of the H1N1 virus was carried out. Optimal inhibition of H1N1 virus pathogenicity by the combination 3′-SL+OPN (MH). Its treatment resulted in no significant difference in the production of inflammatory factors and iNOS production in cells compared to non-viral treated controls (p>0.05), indicating recovery of the inflammatory phenomenon caused by H1N1 virus infection.

### 3.6 RT-qPCR

**Figure 3:**
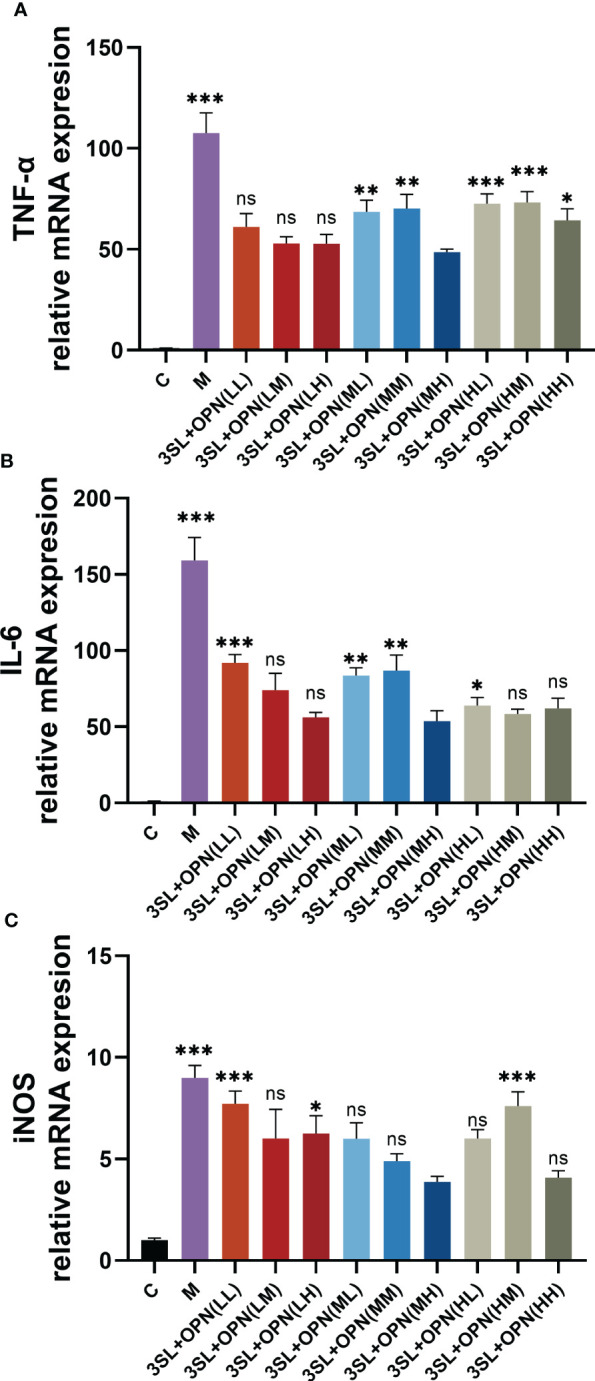
Expression of mRNA for inflammatory factors and iNOS during anti-H1N1 virus by nutrients and their different combinations. The study included the following groups: control group (C), a model group (M), 3′-SL+OPN group (20 μg/mL + 60 μg/mL, LL), (20 μg/mL + 200 μg/mL, LM), (20 μg/mL + 500 μg/mL, LH), (200 μg/mL + 60 μg/mL, ML), (200 μg/mL + 200 μg/mL, MM), (200 μg/mL + 600 μg/mL, MH), (500 μg/mL + 60 μg/mL, HL), (500 μg/mL + 200 μg/mL, HM), and (500 μg/mL + 600 μg/mL, HH). Data are represented as mean ± SD (n= 3). *P< 0.05; **P <0.01; ***P <0.001, ns, not significant, compared with the 3SL+OPN (MH) group.

To further confirm the inhibitory effect of nutrients on the virus, this study also examined the expression of mRNA for inflammatory factors at the gene level using RT-qPCR. The expression of mRNA for inflammatory factors in the control group was very low, while the mRNA expression of the inflammatory factors TNF-a and IL-6 was found to be 100 and 150 times higher, respectively, after viral H1N1 infection (**Figures 3A, B**), and the mRNA expression of iNOS was elevated by about 9 times (**Figure 3C**). A significant reduction in the expression of mRNA for the three inflammatory factors was also found after treatment with both nutrients, particularly in the 3′-SL+OPN (MH) group where the mRNA expression of all three inflammatory factors reached the lowest levels of all groups: TNF-a 48.52 ± 1.53, IL-6 53.27 ± 6.88 and iNOS 3.87 ± 0.28, which is similar to the results for inflammatory factor production.

### 3.7 Inhibition of influenza H1N1 virus by the compositions

**Figure 4:**
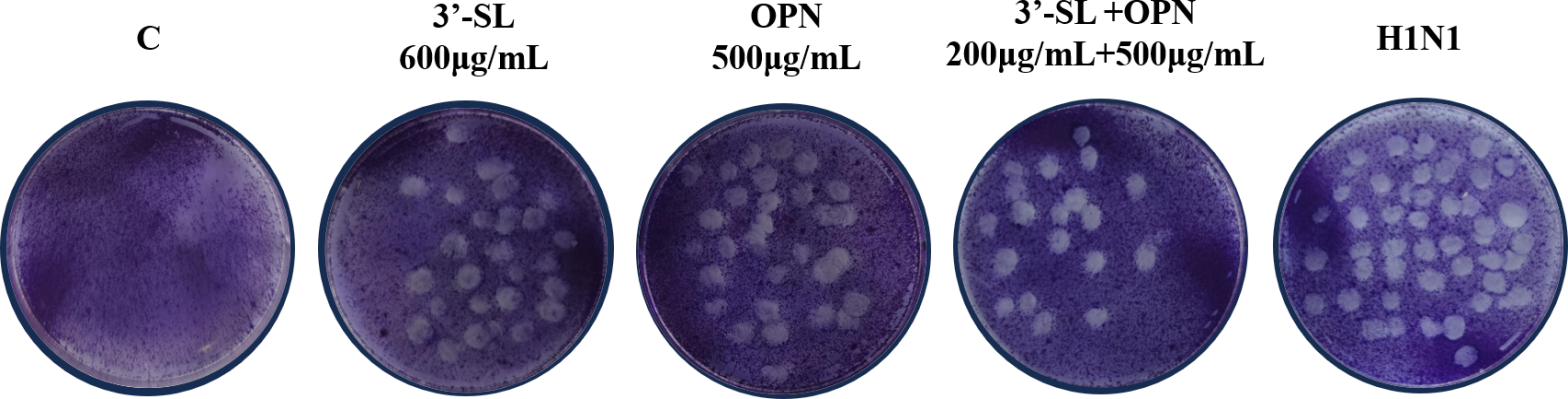
Inhibitory effect of 3′-SL, OPN and 3′-SL+OPN on H1N1 detected by plaque assay.

To confirm the synergistic effect of the combination of 3′-SL+OPN (200 μg/mL+500 μg/mL) in inhibiting viral cytopathogenic lesions, a plaque assay was conducted, and the results are presented in **Figure 4**. The number of plaques was significantly increased in the H1N1 group compared to the non-virus-infected group C. 3′-SL (600 μg/mL) and OPN (500 μg/mL) exhibited an inhibitory effect on viral plaques, but this effect was more significant when the two compounds were combined together. The results suggest that 3′-SL and OPN, especially when combined, had a direct effect on the virus, resulting in a reduction of viral infectivity.

The TCID_50_ assay was used to investigate the reduction of H1N1 virus in HEP-2 cells under nutrient combination intervention. **Table 3** presents the TCID_50_ values for H1N1 virus in different conditions. Without nutrient intervention, the TCID_50_ of H1N1 was 10^-3.71^/0.1 mL. However, when OPN (500 μg/mL) or 3′-SL (600 μg/mL) was administered alone, the H1N1 virus was significantly suppressed, resulting in TCID_50_ values of 10^-3.23^/0.1 mL and 10^-2.9^/0.1 mL, respectively. Remarkably, the combination of OPN and 3′-SL exhibited an even more pronounced suppression of the H1N1 virus, with a TCID_50_ value of 10^-2.65^/0.1 mL.

**Table 3:** Inhibition of Influenza H1N1 Virus by the Compositions.

Nutrients	concentration	TICD_50_(/0.1mL)
3′-SL	600 mg/mL	10^-3.23^
OPN	500 μg/mL	10^-2.9^
3′-SL+ OPN	200 μg/mL+500 μg/mL	10^-2.65^
No Nutrients		10^-3.71^

## 4 Discussion

The H1N1 virus is a common influenza virus that primarily affects the upper respiratory tract in humans and causes a range of symptoms such as fever, body fatigue, sore throat, dry cough and flu (17). The respiratory tract is an important structure connecting the larynx to the lungs and includes the nasal cavity, pharynx, trachea, bronchi and alveolar tissues (18), Therefore, it is crucial to maintain a healthy respiratory tract. Human laryngeal cancer cells are extensively utilized in viral and antiviral drug research due to their relatively high susceptibility to viral infection. The selection of the HEP-2 human laryngeal cancer cell line for this study was based on the fact that it represents the organ site where HMOs and OPNs come into direct contact with the host.

The nutrients used in this study are derived from natural breast milk ingredients and their safety has been scientifically proven in numerous studies. In our experiments, cells came into direct contact with nutrients after infection with the H1N1 virus, and a potential precursor cause could be that the nutrients inhibited the attachment of the virus to the cells, altering the binding efficiency of H1N1 to HEP-2 cells, and thus inhibiting the virus from being effective in causing the cells to become diseased. A recent study has shown that glycosaminoglycans (HM-GAGs) in breast milk prevent cells from binding to cytomegalovirus (19), highlighting the active function of breast milk. In addition, some HMOs can also reduce the binding ability of most pathogens (e.g. Escherichia coli, Vibrio cholerae, Philis Salmonella and Helicobacter pylori) to intestinal epithelial cells *in vitro* (20, 21), thus reducing gastrointestinal diseases. This ability to enhance the antiviral effect by mitigating attachment may be related to the unique structure of nutrients and viruses. For example, the ability of breast milk oligosaccharide 2′-FL to mimic the norovirus receptor, blood group antigen (HBGA), and act as a decoy to prevent norovirus from binding to HBGA (12). HMOs and OPNs may have similar decoy mechanisms to influenza viruses (22). It is worth noting that while the present study only identified 3′-SL as being resistant to H1N1, other studies have indicated that 6′-SL may also possess broad-spectrum antiviral activity. This is attributed to the ability of salivary acidified molecules to bind to crucial influenza virus proteins, specifically the hemagglutinin proteins, and compete with host cells (23). Our data show variability in the inhibitory potency of different structures of HMOs against H1N1 viruses and find that salivary acidified 3-SL exerts the best inhibitory effect, consistent with the previously reported ability of salivary acidification to reduce selectin-mediated leukocyte adhesion (24). This result provides new evidence and insight into the link between the structure of HMOs and their ability to resist viruses. However, the detailed structural mechanisms targeting the attenuation of influenza virus attachment deserve further exploration.

Numerous *in vitro* and *in vivo* experiments have shown that the hyperinflammatory response to influenza virus infection is a key factor in organismal damage (25–27). The TOLL receptor pathway, which triggers inflammatory damage during viral cytopathogenesis, is often thought to be highly associated with increased influenza morbidity and significant changes in TNF-α, IL-6 and iNOS during Toll receptor pathway activation (28, 29). Pro-inflammatory cytokines such as TNF-α and IL- 6 play a key regulatory role in the inflammation-induced immune response (30, 31). Tumor necrosis factor-a (TNF-α) is a pleiotropic cytokine produced by a variety of cells in response to inflammatory and immunomodulatory stimuli and can induce cytopathic regulation (32). IL-6 is a strong activator of the acute phase response, contributing to the systemic and local inflammatory response, and excess IL-6 can induce a variety of chronic inflammatory diseases (33). NO radicals are also critical in inflammatory and immune responses and are synthesized by enzymes such as NOS (eNOS) and iNOS via the l-arginine pathway (34). Under normal physiological conditions, iNOS is dormant in dormant cells; however, under pathological conditions, it produces large amounts of NO and plays a dual role in chronic infections, inflammation (35). Reducing NO production may be an effective strategy for treating a wide range of inflammatory diseases (36). In addition, influenza virus induces proliferation of TNF-α, IL-6, and iNOS, and overexpression of IL-6 and TNF-α promotes influenza virus replication (37), which, together with iNOS, are involved in the hyperinflammatory response to influenza virus infection (38). Breast milk-derived active substances have been reported to exhibit significant inflammatory and immunomodulatory effects both *in vitro* and *in vivo*. Powdered infant formula supplemented with HMOs protects the colon from infection and reduces the inflammatory response by preventing necrotizing small intestinal colitis (NEC) in mice or piglets and by inhibiting activation of the TLR4 signaling NF-κb signaling pathway (39). Moreover, *In vitro*, 2′-FL directly inhibits lipopolysaccharide (LPS)-induced inflammatory responses in intestinal epithelial cells (IECs), decreases IL-8 release, and suppresses transcription and translation of CD14, whose overexpression increased inflammatory responses (40). Recent studies have demonstrated that 2′-FL can enhance immunomodulation and reduce inflammatory responses following influenza vaccination (41). This activity may also have an direct impact on respiratory inflammation caused by influenza viruses. Our data show that TNF-α, IL- 6 and iNOS are increased to varying degrees in HEP-2 cells after H1N1 virus infection. In terms of mRNA expression, the expression of TNF-α and IL- 6 increases up to an alarming 100-fold and 150-fold after H1N1 virus infection, and the excessive inflammatory factors cause inflammatory damage to human respiratory tissues, resulting in discomfort such as sore throat and dry cough. However, after 3′-SL and OPN treatment, the levels and expression of TNF-α, IL- 6 and iNOS were significantly reduced, alleviating cellular inflammation. The plaque assay and TCID_50_ also indicated that lower concentrations of the combined nutrients were more effective in inhibiting the H1N1 virus compared to higher concentrations of single nutrient interventions.

## 5 Conclusion

Overall, our work demonstrated the ability of 3′-SL and OPN to inhibit H1N1 influenza virus cytopathogenesis *in vitro* and found that the most effective combination of antiviral doses was 200 μg/mL of 3′-SL combined with 500 μg/mL of OPN. HMOs and OPN have almost no toxic effect on HEP-2 cells. Acidic breast milk oligosaccharide 3′-SL had a more effective inhibitory effect on the cellular attack of H1N1 virus. When used in combination with OPN, its combined antiviral capacity exceeded the effect of a single substance. This antiviral capacity was associated with a reduction in the inflammatory response in HEP-2 cells, and 3′-SL with OPN was able to significantly reduce the levels and mRNA expression of the cytokines TNF-α, IL-6 and iNOS as a result of H1N1 virus infection, enhancing the innate immunity of cells to H1N1 virus *in vitro*. The synergistic effect of the same phenomenon was observed using both plaque assay and TCID_50_ assay, confirming its effectiveness. However, further exploration is required to understand the mechanism of synergy, along with conducting human clinical studies to validate the findings of the *in vitro* studies.

## Data availability statement

The raw data supporting the conclusions of this article will be made available by the authors, without undue reservation.

## Ethics statement

Ethical approval and written informed consent were not required for the studies on humans in accordance with the local legislation and institutional requirements because only commercially available established cell lines were used.

## Author contributions

ZG: Data curation, Formal analysis, Investigation, Writing – original draft. QX: Data curation, Methodology, Writing – review & editing. QR: Data curation, Methodology, Writing – review & editing. YL: Data curation, Methodology, Writing – review & editing. KL: Data curation, Methodology, Writing – review & editing. BL: Project administration, Writing – review & editing. JL: Project administration, Writing – review & editing.
